# Genetic identification of *Ly75* as a novel quantitative trait gene for resistance to obesity in mice

**DOI:** 10.1038/s41598-018-36073-0

**Published:** 2018-12-05

**Authors:** Keita Makino, Akira Ishikawa

**Affiliations:** Laboratory of Animal Genetics and Breeding, Graduate School of Bioagricultural Sciences, Nagoya University, Chikusa-ku, Nagoya 464-8601 Japan

## Abstract

Identification of causal quantitative trait genes (QTGs) governing obesity is challenging. We previously revealed that the *lymphocyte antigen 75* (*Ly75*) gene with an immune function is a putative QTG for *Pbwg1.5*, a quantitative trait locus (QTL) for resistance to obesity found from wild mice (*Mus musculus castaneus*). The objective of this study was to identify a true QTG for *Pbwg1.5* by a combined approach of a quantitative complementation test, qualitative phenotypic analyses and causal analysis using segregating populations. In a four-way cross population among an *Ly75* knockout strain, a subcongenic strain carrying *Pbwg1.5* and their background strains, the quantitative complementation test showed genetic evidence that the *Ly75* locus is identical to *Pbwg1.5*. Qualitative phenotypic analyses in two intercross populations between knockout and background strains and between subcongenic and background strains suggested that *Ly75* may have pleiotropic effects on weights of white fat pads and organs. Causal analysis in the intercross population between knockout and background strains revealed that only variation in fat pad weight is caused by the genotypic difference via the difference in liver *Ly75* expression. The results showed that *Ly75* is a true *Pbwg1.5* QTG for resistance to obesity. The finding provides a novel insight for obesity biology.

## Introduction

Obesity is a major risk factor for type 2 diabetes mellitus, hypertension, cardiovascular diseases, some cancers and other health problems^1^. It is intricately controlled by many genetic loci, called quantitative trait loci (QTLs), environmental factors and their interactions. Many QTLs for obesity traits have been identified by genome-wide association studies (GWASs) in humans^2^, though the QTLs identified explain only a small fraction of the genetic variation^3^, suggesting that many QTLs with small effects on obesity remain unidentified. Likewise, in mice, many obesity QTLs have been mapped to chromosomal regions across most of the genome^4^. However, except for a few successful examples^5,6^, it has been difficult to identify causal genes, called quantitative trait genes (QTGs), for common QTLs with small effects on obesity. Identification of additional QTGs will provide new insights for obesity biology.

We previously revealed many QTLs for postnatal body weight and growth using an undeveloped resource of wild mice (*Mus musculus castaneus*) in the Philippines by genome-wide QTL analysis in an intersubspecific backcross population between the wild mice and the C57BL/6JJcl (B6JJcl) inbred strain being prone to obesity and type 2 diabetes mellitus^7,8^. We created an original congenic strain carrying *postnatal body weight growth 1* (*Pbwg1*), a major QTL for growth on mouse chromosome 2, on the B6JJcl genetic background and we named the strain B6.Cg-*Pbwg1* (Supplementary Fig. S1). *Pbwg1* increases its phenotypic effect linearly with increasing age and explains approximately 4–12% of the total phenotypic variance depending on the age examined^8^. By fine mapping of *Pbwg1* using B6.Cg-*Pbwg1* and subcongenic strains developed from B6.Cg-*Pbwg1*, we identified a QTL (*Pbwg1.5*) for the weight of white fat pads^9^. The wild-derived allele at *Pbwg1.5* uniquely decreased fat pad weight and showed resistance to obesity in mice fed low-fat and high-fat diets^10^. Exome and bioinformatics analyses prioritized *integrin beta 6* (*Itgb6*) and *lymphocyte antigen 75* (*Ly75*) as presumable candidate genes for *Pbwg1.5*^11^. Furthermore, an integrated approach of mRNA expression analysis and causal analysis provided statistical evidence that only liver *Ly75* expression mediates between genotype and white fat pad weight, suggesting that *Ly75* with an immune function is a putative QTG for *Pbwg1.5*^12^. In the present study, we obtained genetic evidence that *Ly75* is a true QTG for the *Pbwg1.5* QTL for resistance to obesity by using a combined approach of a quantitative complementation test, qualitative phenotypic analyses and causal analysis in segregating populations obtained from crosses using the B6.129P-*Ly75*^*tm/Mnz*^/J strain knocked out for *Ly75* (hereafter called KO)^13^, the B6.Cg-*Pbwg1*/24Nga subcongenic strain carrying *Pbwg1.5* (SR24; Supplementary Fig. S1) and their background strains of C57BL/6 J (B6J) and B6JJcl.

## Results

### DNA sequencing of the KO gene

The KO strain for *Ly75* was previously created by disrupting the promoter and exon 1 of *Ly75* by a targeting vector containing a neomycin resistance gene^13^. By sequence analysis of genomic DNA of the strain, we confirmed this disruption and found that both the deletion of *Ly75* exon 1 and the insertion of the neomycin resistance gene resulted in a frameshift mutation leading to creation of a new stop codon in *Ly75* exon 2 (Supplementary Fig. S2).

### Phenotypic analysis in progenitor strains

Thirty quantitative traits including food intakes, body weights, body lengths and weights of white fat pads and organs were measured in male mice of four progenitor strains, KO, B6J, SR24 and B6JJcl (Supplementary Table S1). KO mice had significantly longer total body lengths and tail lengths and higher liver and kidney weights than those of the background B6J mice at *P* < 0.05 (t-test). These traits adjusted for body weight at 14 weeks of age (hereafter called adjusted traits) were also significantly different between KO and B6J mice. However, no significant differences in any weights of white fat pads were observed between KO and B6J strains. By contrast, SR24 mice had significantly lower unadjusted and adjusted weights of total and gonadal white fat pads and higher unadjusted and adjusted weights of heart and kidneys than those of the background B6JJcl mice at *P* < 0.05. These results confirmed that the SR24 strain has the *Pbwg1.5* QTL and suggested that the KO strain may not display obese phenotype.

### Quantitative complementation test

**Figure 1:**
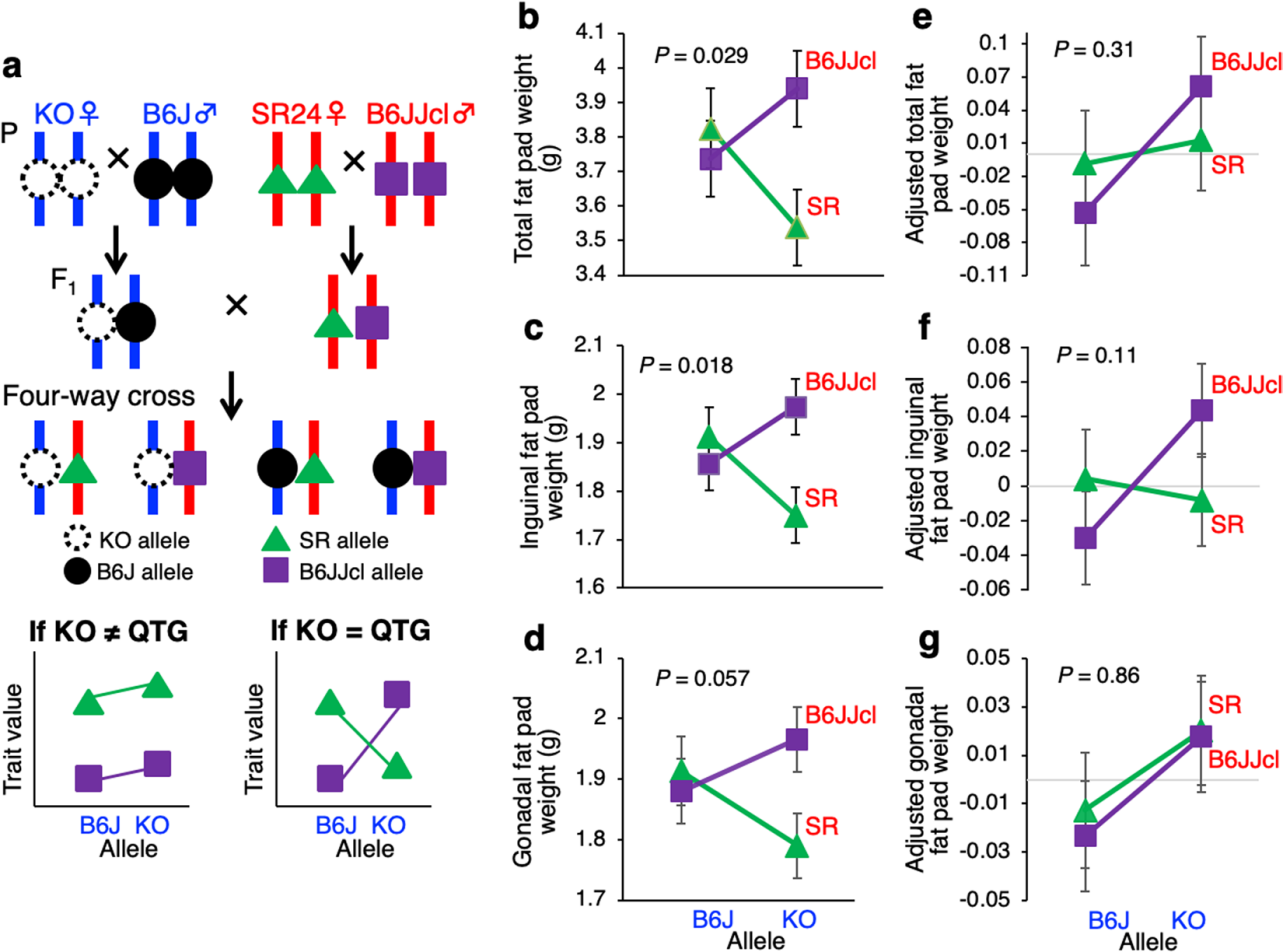
Quantitative complementation test. (**a**) Mating design of a four-way population among the B6.129P-*Ly75*^*tm/Mnz*^/J (KO) strain knocked out for the *Ly75* gene, its background C57BL/6 J (B6J) strain, the SR24 subcongenic strain carrying the *Pbwg1.5* QTL (see Supplementary Fig. S1) and its background C57BL6/JJcl (B6JJcl) strain. The former two strains have all of the same chromosomes (blue vertical bars) except for the congenic region of the129P2/OlaHsd strain in which alleles at some loci around the *Ly75* locus may be different between KO and B6J strains. The latter two strains have the same chromosomes (red vertical bars) except for the subcongenic region in which alleles at some loci may be different between SR24 and B6JJcl strains. In the four-way cross population, four possible genotypes are segregating on a uniform genetic background. No statistical interaction between KO and QTL alleles means that the *Ly75* locus is not equal to the QTL, whereas a significant interaction indicates that these loci are the same. (**b**–**d**) Weights of white fat pads, unadjusted for body weight at 16 weeks of age. (**e**–**g**) Adjusted weights of white fat pads, adjusted for the body weight. The overall mean of adjusted weights for all genotypes was set to zero. *P* values were obtained from two-way ANOVA. Detailed data for all traits measured are shown in Supplementary Table S2.

A quantitative complementation test is a mating experiment to determine whether the locus of a gene knocked out is the same as a QTL for a given trait by testing an interaction effect between KO and QTL alleles by two-way analysis of variance (ANOVA)^14^. To carry out the quantitative complementation test on a uniform genetic background, we made a four-way cross among the KO strain and the SR24 subcongenic strain and their corresponding background strains of B6J and B6JJcl, as shown in Fig. 1a. In the four-way cross population obtained, mice with four possible genotypes of B6J/B6JJcl, B6J/SR24, KO/B6JJcl and KO/SR24 were segregating. Here, no significant interaction effect between KO and QTL alleles (i.e., quantitative complement) is interpreted as evidence that the KO locus in the B6J strain is not equal to the QTL in the SR24 strain. In contrast, a significant interaction (i.e., quantitative failure to complement) is interpreted as evidence that the KO locus is identical to the QTL (Fig. 1a).

Thirty-five traits for body weight, body weight gain, body lengths, fat pad weights and organ weights were measured in 352 mice (182 males and 170 females) of the four-way cross population obtained (Supplementary Tables S2). Except for total body lengths, total fat pad weights and testis weights, the remaining 29 traits were classified into eight trait groups by category to perform multivariate analysis of variance (MANOVA) for the eight trait groups separately. The eight trait groups were for body weight, body weight gain, body length, organ weight, fat pad weight, adjusted body length, adjusted organ weight and adjusted fat weight. As shown in Supplementary Table S3, MANOVA identified significant interaction effects between QTL and KO alleles for two trait groups of fat pad weight and adjusted organ weight at *P* < 0.05 (F-test). No KO-by-QTL-by-sex interactions were observed for all trait groups. Hence, using sex-combined data, two-way ANOVA were performed for each trait separately. Two-way ANOVA revealed significant interaction effects between QTL and KO alleles for body weight at 16 weeks of age, body weight gain at 13–16 weeks, total fat pad weight, inguinal fat pad weight and adjusted liver weight at *P* < 0.05 (Supplementary Tables S2, Fig. 1b,c). The interaction effect was marginal for gonadal fat pad weight at *P* = 0.057 (Fig. 1d). Although the interaction effect was not significant for any adjusted fat pad weights, a tendency for the interaction was observed for adjusted weights of total and inguinal fat pads (Fig. 1e–g). These results suggested that the locus of the KO gene, i.e., *Ly75*, is identical to the *Pbwg1.5* QTL and that *Pbwg1.5* may have pleiotropic effects on body weight, weight gain and liver weight.

### Qualitative phenotypic analyses

**Figure 2:**
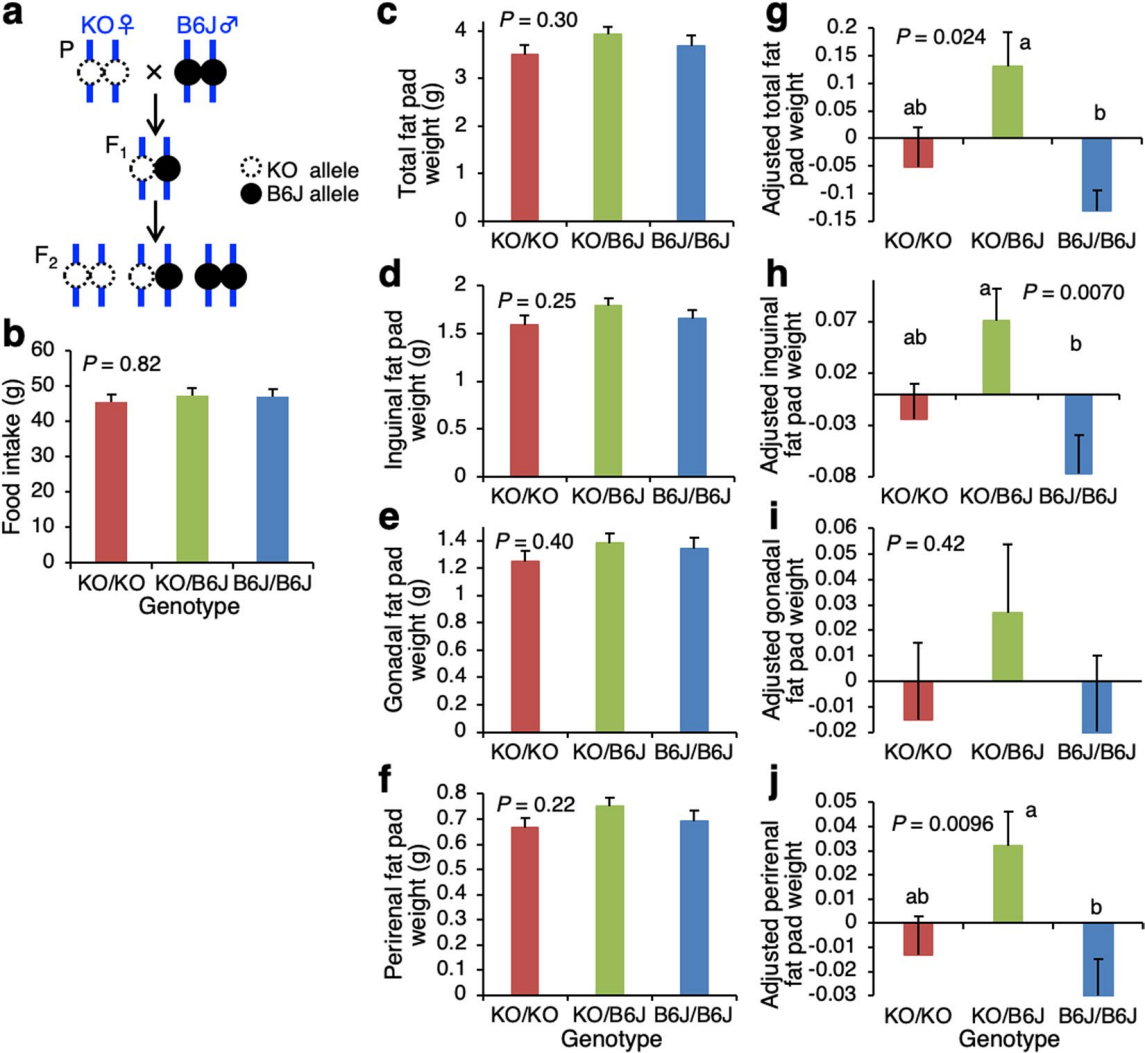
Qualitative phenotypic analyses. (**a**) Mating design of an F_2_ intercross population between KO and B6J strains, in which three possible genotypes are segregating. (**b**) Food intake for three males per genotype. (**c**–**f**) Weights of white fat pads. (**g**–**j**) Adjusted weights of white fat pads. The overall mean of adjusted weights for all genotypes was set to zero. *P* values were obtained from one-way ANOVA. Least squares means with different superscript letters (a,b) indicate significant differences in a trait between the three genotypes at *P* < 0.05 (Tukey’s HSD test). Detailed data for all traits measured are shown in Supplementary Table S4.

To confirm whether the pleiotropic effects detected by the above complementation test are present, we developed an F_2_ population by intercrossing between KO and B6J strains, in which three possible genotypes of KO/KO, KO/B6J and B6J/B6J were segregating as shown in Fig. 2a. Amounts of food intake were not significantly different among mice with the three genotypes at *P* = 0.82 (one-way ANOVA) fed a standard chow for a two-week period from 4 to 6 weeks of age (Fig. 2b). In total, 37 traits including body weights and organ weights were measured in 108 F_2_ mice (67 males and 41 females) (Supplementary Table S4). MANOVA of eight trait groups identified significant genotypic effects for four groups of body length, organ weight, adjusted organ weight and adjusted fat pad weight at *P* < 0.05 (at least Roy’s Maximum Root). No genotype-by-sex interaction effect was identified for all trait groups (Supplementary Table S5). Using sex-combined data, one-way ANOVA were performed for each trait separately. One-way ANOVA identified significant genotypic effects on two organ weights and five adjusted organ weights at *P* < 0.05 (Supplementary Table S4). Although one-way ANOVA identified no significant genotypic effects on all fat pad weights and adjusted gonadal fat pad weight, it identified significant effects on three adjusted fat pad weights at *P* = 0.0070–0.024 (Supplementary Table S4, Fig. 2c–j). All fat and organ traits for KO/KO mice were not significantly different from those of KO/B6J and/or B6J/B6J mice at *P* > 0.05 (Tukey’s honestly significant difference (HSD) test) (Supplementary Table S4). On the other hand, B6J/B6J mice showed significantly lower adjusted weights of total, inguinal and perirenal fat pads than those of KO/B6J mice at *P* < 0.05 (Fig. 2g,h,j). In contrast, B6J/B6J mice showed significantly higher unadjusted and adjusted weights of spleen and adjusted weights of kidneys, heart and lungs than those of KO/B6J mice at *P* < 0.05 (Supplementary Table S4). These results suggested that the B6J allele at the *Ly75* locus may exert its effect pleiotropically on weights of fat pads, spleen, kidneys, heart and lungs.

To further confirm the pleiotropic effects, 38 traits were measured in a different population of 91 F_2_ male mice obtained from an intercross between KO and SR24 strains (Supplementary Table S6). Food intake was not significantly different among mice with three genotypes of KO/KO, KO/SR and SR/SR at *P* = 0.90 (one-way ANOVA). MANOVA of eight trait groups identified significant genotypic effects on four trait groups of body weight, organ weight, adjusted organ weight and adjusted fat pad weight at *P* < 0.05 (at least Roy’s Maximum Root) (Supplementary Table S7). One-way ANOVA of individual traits in the four groups revealed significant genotypic effects on weights of fat pads and organs at *P* < 0.05 (Supplementary Table S6). All fat and organ traits of KO/KO mice were not significantly different from those of KO/B6J mice at *P* > 0.05 (Tukey’s HSD test) By contrast, adjusted weights of total, gonadal and perirenal fat pads for SR/SR mice were significantly lower than those of KO/KO and/or KO/SR mice at *P* < 0.05 (Tukey’s HSD test). However, unadjusted and adjusted weights of kidneys and unadjusted heart weight for SR/SR mice were significantly higher than those of KO/KO and/or KO/SR mice at *P* < 0.05 (Tukey’s HSD test). These results suggested that the SR allele at the *Ly75* locus may exert its effect pleiotropically on weights of fat pads, kidneys and heart.

Taken all together, the above results in the two F_2_ populations suggested that the *Ly75* locus may have pleiotropic effects on weights of fat pads, kidneys, heart and other organs. Furthermore, the results suggested that KO/KO mice may not have obvious obese phenotypes.

### Causal analysis

**Figure 3:**
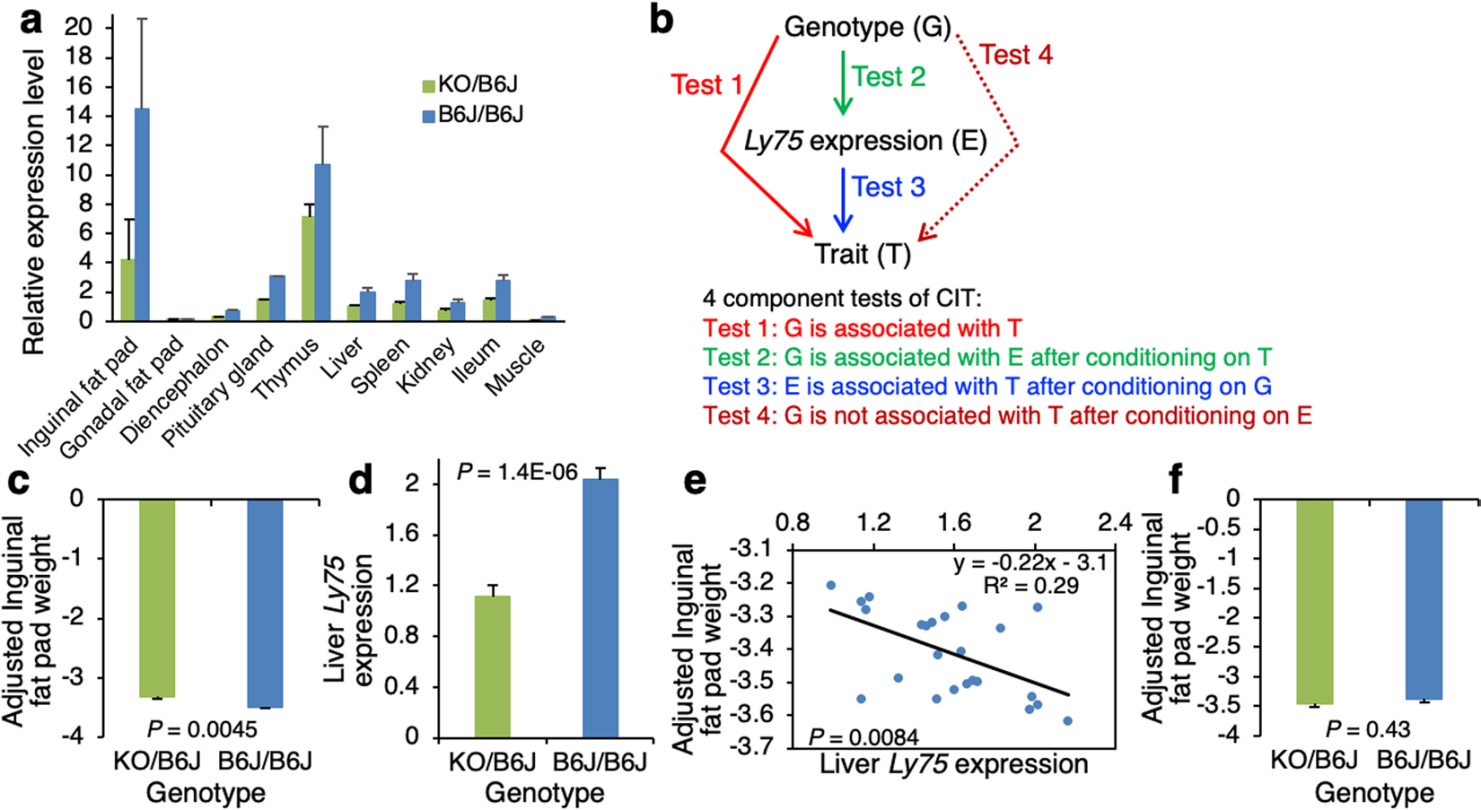
Tissue expression pattern of *Ly75* and the causal inference test (CIT) of adjusted inguinal fat pad weight in KO/B6J and B6J/B6J mice of an F_2_ population between KO and B6J strains. (**a**) *Ly75* expression in 10 tissues. Data are means ± S.E.M. for expression levels relative to the KO/B6J liver. Detailed data are shown in Supplementary Table S8. (**b**) Criteria for CIT^16^. Four component tests of CIT assess whether the change in genotype (G) leads to variation in a quantitative trait (T) via the change in *Ly75* expression (E). (**c**) Result of Test 1. (**d**) Result of Test 2. (**e**) Result of Test 3. (**f**) Result of Test 4. In (**c**–**f**), data are least squares means ± S.E.M. *P* values were obtained from linear regression models of the component tests shown in (**b**).

Before causal analysis, we investigated the tissue expression pattern of the *Ly75* gene by quantitative real-time PCR analysis in KO/B6J and B6J/B6J male mice (n = 3 for each genotype) of the F_2_ population between KO and B6J strains. *Ly75* was highly expressed in the thymus, a target tissue of *Ly75*, and the inguinal fat pad of both KO/B6J and B6J/B6J mice (Supplementary Table S8, Fig. 3a). *Ly75* expression levels in the diencephalon, pituitary gland, thymus, liver, spleen, kidney and ileum of B6J/B6J mice were approximately twice as high as KO/B6J mice, reflecting the difference in copy number of the B6J-derived *Ly75* gene between the KO/B6J and B6J/B6J mice, as expected. However, inguinal and gonadal fat pads and gastrocnemius muscles showed unexpected expression differences of one or more than three folds, though a further confirmation study is needed. As the liver is a main organ for lipid metabolism^15^, we used liver *Ly75* expression levels for the causal inference test (CIT), a causal analysis that can infer causal relationships between genotype, gene expression and trait^16^.

To find a genuine trait(s) affected by *Ly75* from the fat and organ traits identified by the above quantitative complementation test and qualitative phenotypic analyses, we performed four component tests of the CIT (Fig. 3b) for 20 traits of fat and organ weights measured, and the results are summarized in Table 1. For Test 1, the genotype was significantly associated with 11 traits of fat and organ weights at *P* = 0.00057–0.024 (t-test). For Test 2, the genotype was highly and significantly associated with liver *Ly75* expression after conditioning on each of the traits. For Test 3 that conditioned on the genotype, liver *Ly75* expression was significantly associated with adjusted weights of total and inguinal fat pads at *P* = 0.0084–0.022. In addition, *Ly75* expression was marginally associated with unadjusted weights of total, inguinal and gonadal fat pads at *P* = 0.055–0.081. Interestingly, *Ly75* expression was not significantly associated with unadjusted and adjusted weights of perirenal fat pads as well as adjusted weights of kidneys, heart, lungs and spleen. For Test 4 that conditioned on liver *Ly75* expression, the genotype was independent of adjusted and unadjusted weights of total and inguinal fat pads.

**Table 1:** Results of CIT for fat pad weights and organ weights of male mice obtained from an F_2_ intercross population between KO and B6J strains.

Trait	Genotype		*P* value				Relationship model^a^
	KO/B6J	B6J/B6J	Test 1	Test 2	Test 3	Test 4	
No. of mice	12	12					
Liver *Ly75* expression level	1.00 ± 0.09	2.13 ± 0.09		1.6E-08			
Total fat pad weight (g)	5.258 ± 0.185	4.580 ± 0.185	0.017	5.7E-07	0.081^b^	0.76	Causal^b^
Inguinal fat pad weight (g)	2.416 ± 0.093	2.065 ± 0.093	0.014	6.6E-07	0.061^b^	0.71	Causal^b^
Gonadal fat pad weight (g)	1.860 ± 0.068	1.675 ± 0.068	0.068	1.3E-07	0.055	0.43	
Perirenal fat pad weight (g)	0.981 ± 0.033	0.840 ± 0.033	0.0063	9.7E-07	0.54	0.39	
Liver weight (g)	1.158 ± 0.025	1.149 ± 0.025	0.82	2.7E-08	0.32	0.44	
Kidney weight (g)	0.357 ± 0.007	0.377 ± 0.007	0.051	1.9E-07	0.73	0.51	
Heart weight (g)	0.117 ± 0.002	0.121 ± 0.002	0.28	5.8E-08	0.90	0.69	
Lung weight (g)	0.138 ± 0.002	0.144 ± 0.002	0.024	2.6E-07	0.88	0.33	
Spleen weight (g)	0.076 ± 0.034	0.086 ± 0.034	0.050	2.3E-07	0.43	0.78	
Testis weight (g)	0.218 ± 0.015	0.196 ± 0.015	0.31	2.0E-08	0.26	0.15	
Adjusted total fat pad weight	−6.112 ± 0.077	−6.433 ± 0.077	0.0074	1.0E-06	0.022	0.55	Causal
Adjusted inguinal fat pad weight	−3.326 ± 0.038	−3.497 ± 0.038	0.0045	1.4E-06	0.008	0.43	Causal
Adjusted gonadal fat pad weight	−1.672 ± 0.042	−1.747 ± 0.042	0.22	3.6E-08	0.047	0.24	
Adjusted perirenal fat pad weight	−1.114 ± 0.013	−1.189 ± 0.013	5.7E-04	1.1E-06	0.61	0.029	
Adjusted liver weight	0.067 ± 0.016	0.093 ± 0.016	0.27	3.5E-08	0.52	0.28	
Adjusted kidney weight	0.146 ± 0.005	0.173 ± 0.005	0.0018	3.1E-06	0.34	0.42	
Adjusted heart weight	0.055 ± 0.002	0.060 ± 0.002	0.047	2.2E-07	0.55	0.65	
Adjusted lung weight	0.119 ± 0.002	0.126 ± 0.002	0.011	5.1E-07	0.76	0.31	
Adjusted spleen weight	0.016 ± 0.003	0.028 ± 0.003	0.013	7.1E-07	0.26	0.78	
Adjusted testis weight	−0.034 ± 0.015	−0.049 ± 0.015	0.50	1.3E-08	0.17	0.13	

Data for two genotypes are presented as the least squares mean ± S.E.M. adjusted for a litter effect. Adjusted data were obtained by adjustment of the raw data by a litter effect and body weight at 16 weeks of age. Total fat pad weight is the sum of weights of inguinal, gonadal and perirenal fat pads. The *P* value for the liver *Ly75* expression level shown in the table was obtained by a simple association analysis without conditioning on any trait values. See Fig. 3b for detailed methods of Tests 1 to 4. ^a^The causal relationship model indicates that the genotypic difference causes the trait difference via the *Ly75* expression difference. ^b^The *P* value is marginal.

Taken together, the above results showed that at least inguinal fat pad weight is the only trait in the causal path. The results of all four CIT tests for the inguinal fat trait are shown in Fig. 3c–f. Liver *Ly75* expression was negatively correlated with adjusted inguinal fat pad weight, and it accounted for approximately 29% of total variation in the fat trait (R^2^ = 0.29).

## Discussion

In this study, we used segregating populations in the quantitative complementation tests and qualitative phenotypic analyses in order to reduce environmental effects, such as litter size, maternal genetic effects and epigenetic effects, on traits examined as much as possible by statistical adjustments for the environmental effects and to randomize the effects of genes located on the contaminated genomic regions from donor strains during development of KO and SR24 congenic strains, as described previously^11,17^. However, almost intact phenotypes were observed in KO/KO mice in the two F_2_ populations between KO and B6J strains and between KO and SR24 strains. This lack of knockout phenotypes may be due to genetic compensation, a phenomenon that functionally compensates for the loss of function of a gene knocked out by another related gene(s)^18^. A recent study in zebrafish showed that knockdown of a skeletal actin gene causes nemaline myopathy characterized by reduced muscle function, whereas the knockout mutation exhibits no obvious muscle damage^19^. In the zebrafish study, it was shown that the muscle damage caused by the knockout mutation is compensated by transcriptional upregulation of an actin paralogue that restores actin protein in the skeletal muscle. As 46 *Ly*-related genes have been reported in mice^4^, the lack of knockout phenotypes seen in our KO/KO mice might be compensated by another *Ly*-related gene or other genes in the same or a similar biological network. In addition, an unexpected obese phenotype in the KO/B6J mice, being seemingly overdominat inheritance, might be due to hemizygosity for the *Ly75* gene, because the obese phenotype shows an additive mode of inheritance in our previous studies using an F_2_ population between the SR1 subcongenic strain carrying the wild-derived *Pbwg1.5* allele (Supplementary Fig. S1) and its B6JJcl background strain^11,12^. We need further transcriptional analysis of the *Ly*-related genes to prove the possibilities.

In mice, the quantitative complementation test using a knockout strain has often been used to determine whether a candidate gene is a true QTG for a given trait^20–22^. In a mouse cross between DBA/2 J and C57BL/6 J strains, the *pregnancy-associated plasma protein A2* (*Pappa2*) gene was shown to be a QTG for a QTL with a small phenotypic effect on body size including tail length, bone length and body weight^21^. However, that study showed that an interaction effect between *Pappa2* and QTL alleles was significantly different only in tail length and body weight at 3 weeks of age, while it was marginally different in lengths of the skull and long bones and body weight at 6 weeks and 10 weeks. Hence, that study showed that, even if the locus of a gene that has been knocked out is the same as a QTL having small phenotypic effects, interaction effects would not always reach statistical significant levels. The results of that study agree with our results showing that an interaction effect between KO and QTL alleles was significant for total and inguinal fat pad weights, whereas it was marginal for gonadal fat pad weight and adjusted weights of total and inguinal fat pads. We finally confirmed a significant effect of *Ly75* on fat weight traits by qualitative phenotypic analyses using two different F_2_ populations between KO and B6J strains and between KO and SR24 strains.

Our quantitative complementation test and qualitative phenotypic analyses suggested that *Ly75* may have pleiotropic effects on fat and organ weight traits. Among these traits, CIT analysis successfully revealed at least adjusted inguinal fat pad weight as a genuine trait, variation in which was caused by *Ly75* genotypes via *Ly75* expression. The CIT analysis was able to exclude retroperitoneal fat and organ traits from the genuine trait. Likewise, in a mouse intercross between C57BL/6ByJ and 129P3/J strains, it was shown that a QTL on chromosome 9 affects % weight of gonadal fat depots but does not affect that of retroperitoneal fat depots^23^. The presence of such fat-pad-specific QTLs is no surprise, since each fat pad can have a unique expression pattern of developmental genes such as homeobox genes^24^. Therefore, it is strongly suggested that *Ly75* has no pleiotropic effects on organ weights and has a causal effect on inguinal fat, in which *Ly75* was highly expressed in the present study.

In the quantitative complementation test and qualitative phenotypic analyses, we used the KO strain, i.e., B6.129P-*Ly75*^*tm/Mnz*^/J, which is a congenic strain developed by introgression of a 129 P genomic region carrying the disrupted *Ly75* gene into the B6J genetic background. Hence, the possibility cannot be ruled out that the genes closely linked to the disrupted *Ly75* gene on the 129 P congenic region affected our results. In our previous study using a different F_2_ population between the SR1 subcongenic strain and the B6JJcl strain, several closely-linked genes on the SR1 congenic region were differentially expressed^12^. In the previous study, CIT analysis successfully excluded the closely-linked genes from candidate genes for *Pbwg1.5*. As very recently reviewed^17,25^, CIT analysis is an essential methodology for seeking out any genuine factor involved in the causal link between genetic variation and trait variation from confounding factors. In the present study, we used only *Ly75* expression for CIT analysis. It is therefore unlikely that the closely-linked genes on the 129 P congenic region affected the CIT results in the present study.

Our previous study in the F_2_ population between SR1 and B6JJcl strains showed that mice homozygous for the wild-derived SR1 region had an approximately three-fold higher level of *Ly75* expression in the liver than that in B6JJcl/B6JJcl mice and that the expression was negatively correlated with fat pad weight^12^. Hence, we can hypothesize that if the level of *Ly75* expression is the only essential factor affecting fat weight, mice with a higher *Ly75* expression level will have lower fat weight than that of mice with a lower expression level irrespective of the origin in the *Ly75* allele derived from either a B6JJcl or wild mouse. This hypothesis is validated by the results of the present study using the F_2_ population between KO and B6J strains showing that the fat weight of B6J/B6J mice with an approximately two-fold higher level of *Ly75* expression was significantly lower than that of KO/B6J mice with an approximately one-fold higher expression level. Both KO/B6J and B6J/B6J mice have the same *Ly75* gene derived from a single B6J male (see Methods), without differences in DNA sequences of the genes between the KO/B6J and B6J/B6J mice. Furthermore, replacement of the *Ly75* allele from wild to B6J in the present study provided experimental evidence supporting the previous prediction that nine nonsynonymous single-nucleotide polymorphisms detected by exome sequencing of the *Ly75* gene are not harmful to protein function^11^.

*Ly75* encodes dendritic and epithelial cells, 205 kDa (DEC-205), which is an integral membrane protein homologous to the macrophage mannose receptor, and DEC-205 acts as an endocytic receptor to direct captured antigens from the extracellular space to a specialized antigen-processing compartment^26^. It has been reported that KO mice for *Ly75* exhibit an absence of a cytotoxic lymphocyte response to immunization with foreign splenocytes and have no functional gene product in activated T cells^13^. However, little is known about the direct effect of *Ly75* on fat. Thus, the present study showed the first genetic evidence that *Ly75* is involved in fat regulation.

Until now, *Ly75* has not been reported as a core gene underlying obesity. As we mentioned earlier, QTLs identified by human GWASs explain only a small fraction of the genetic variation in obesity^3^, suggesting that many QTLs explaining most of the genetic variation remain unidentified. This genetic architecture of obesity may exactly match the omnigenic model of genetic architecture^27^. In the omnigenic model, it is thought that many non-core (or peripheral) genes expressed in tissue(s) relevant to disease phenotypes can essentially affect all downstream phenotypes and that the non-core genes do not play direct roles in the disease but contribute to a large fraction of the genetic variation. As shown in the present study and our previous study^12^, *Ly75* is expressed in the mouse liver and fat, which are relevant to obesity. Thus, we conclude that *Ly75* is surely a non-core gene involved in the omnigenic architecture of obesity. Our finding provides a novel insight for obesity biology.

## Methods

### Animals

The SR24 subcongenic strain with a wild-derived *Pbwg1.5* region of approximately 3 Mb (Supplementary Fig. S1) was previously created from descendants of the original B6.Cg-*Pbwg1* congenic strain in our laboratory^17^. Its B6JJcl background strain was purchased from Clea Japan, Inc. (Tokyo, Japan). The *Ly75* KO strain, i.e., B6.129P-*Ly75*^*tm/Mnz*^/J (JAX stock #005528), and its B6J background strain were purchased from The Jackson Laboratory (Maine, USA) through Charles River Laboratories Japan, Inc. (Yokohama, Japan). All mice used in this study were weaned at 3 weeks after birth. The mice were provided a standard chow (CA-1, Clea Japan, Inc.), containing 5% crude fat and 3.5 kcal/g energy, and tap water *ad libitum*, except for quantitative complementation tests and qualitative phenotypic analyses in which high-fat diets (D12451, Research Diets, Inc., New Brunswick, USA), containing 24% fat (45 kcal% fat), were supplied for a few weeks in an attempt to enhance differences in obesity traits among mice with different genotypes (see below). All animal experiments described here were approved by the Animal Research Committee at the Graduate School of Bioagricultural Sciences, Nagoya University and were performed in accordance with the committee’s guidelines.

### DNA sequencing

Genomic DNA was extracted by a standard method from ear clips of the KO mice. The genomic region targeted by a vector containing a neomycin resistance gene^13^ was amplified by primers listed in Supplementary Table S9 and was sequenced using ABI PRISM 3130 Genetic Analyzer (Life Technologies Japan Ltd., Tokyo). DNA sequence data obtained were compared with the RefSeq mm10 of B6J.

### Phenotypic analysis in progenitor strains

Male mice of four progenitor strains, KO (n = 6), B6J (n = 4), SR24 (n = 5) and B6JJcl (n = 6) at 3–14 weeks of age were housed individually in cages in which the mice had free access to tap water and the powder diet of CA-1. Food intake and body weight were measured every two or three days for 14 days between 6 and 8 weeks of age and for 14 days between 12 and 14 weeks of age. Body weight gains at 6–8 weeks and 12–14 weeks of age were calculated. After four hours fasting, mice were killed at 14 weeks of age under isoflurane anesthesia. Total body length, tail length and head-body length were measured as described previously^11^. The heart, lungs, spleen, liver, kidneys, testes, inguinal white fat pad and gonadal white fat pad were weighed.

### Quantitative complementation tests

According to a previous report^17^, three KO females were crossed to two B6J males, and three SR24 females were crossed to two B6JJcl males. Two types of F_1_ mice obtained from these two crosses were reciprocally crossed to each other to produce a four-way cross population. In the four-way-cross mice obtained, four possible genotypes of B6J/B6JJcl (n = 81), B6J/SR (n = 90), KO/B6JJcl (n = 91) and KO/SR (n = 90) were segregating.

The mice were fed a solid high-fat diet at 10–16 weeks of age. Body weights were measured at 1, 3, 6, 10, 13 and 16 weeks of age. Body weight gains at 1–3 weeks, 3–6 weeks, 6–10 weeks, 10–13 weeks and 13–16 weeks of age were calculated. The mice were killed at 16 weeks of age, and body composition traits were measured as described above.

### Qualitative phenotypic analyses

A male of the B6J strain was intercrossed to two females of the KO strain to produce an F_2_ population, in which F_2_ mice with three genotypes of KO/KO (n = 34), KO/B6J (n = 44) and B6J/B6J (n = 30) were segregating. In addition, an F_2_ population of male mice was developed from an intercross between a SR24 male and two KO females. In the F_2_ males obtained, three genotypes of KO/KO (n = 26), KO/SR (n = 38) and SR/SR (n = 27) were segregating. In both F_2_ populations, only male mice between KO and B6J strains (n = 3 per genotype) and between SR24 and B6JJcl strains (n = 6–9 per genotype) were used to measure food intake for 14 days between 4 and 6 weeks of age, as described above.

All F_2_ mice in both populations were fed a high-fat diet from 6 to 16 weeks of age. The mice were dissected at 16 weeks of age, and body weight and body composition traits were measured as described above. The weight of perirenal white fat pads was additionally recorded.

### Genotyping

To determine genotypes of mice obtained for quantitative complementation tests and qualitative analyses described above, genomic DNAs were extracted from ear clips of the mice obtained. Genotypes of the SR24 subcongenic region were determined by PCR amplifications, as described previously^9^, using the *D2Mit123* microsatellite marker and the *rs48690987* SNP marker (Supplementary Table S9). Genotypes at the KO locus were determined using two pairs of PCR primers (Supplementary Table S9) as described in the website of The Jackson Laboratory (https://www2.jax.org/protocolsdb/f?p=116:5:0::NO:5:P5_MASTER_PROTOCOL_ID,P5_JRS_CODE:22921,005528). Recombinant F_2_ individuals were excluded from the present study.

### Quantitative real-time PCR analysis

For tissue expression analysis, total RNA was extracted from 10 tissues of diencephalons, pituitary glands, thymuses, livers, spleens, kidneys, ileums, inguinal fat pads, gonadal fat pads and gastrocnemius muscles of KO/B6J and littermate B6J/B6J male mice (n = 3 per genotype) in the F_2_ population between KO and B6J strains by using Trizol according to the manufacturer’s instructions. For CIT analysis, total RNA was extracted from livers of KO/B6J and littermate B6J/B6J males (n = 12 per genotype). Quantitative real-time PCR analysis was carried out on a StepOnePlus Real-Time PCR system (Thermo Fisher Scientific, Tokyo) with SYBR Premix Ex Taq™ II (Tli RNaseH Plus) (Takara Bio, Otsu, Japan), as described previously^12^. Primer sequences for *Ly75* and an endogenous control gene, *Actb* (actin, beta), are listed in Supplementary Table S9. All samples were analysed in duplicate for the tissue analysis and triplicate for the CIT analysis. The expression levels of *Ly75* were normalized to that of *Actb* and measured using the 2^−ΔΔCT^ method.

### Causal inference test (CIT)

Using the F_2_ males between KO and B6J strains, the causal relationships between KO/B6J and B6J/B6J genotypes (G), *Ly75* expression (E) and phenotypic traits (T) were assessed by four component tests of CIT^16,17^, as shown in Fig. 3b.

### Statistical analysis

Data for body weights, weight gains and body compositions were analysed with a linear mixed model of the statistical discovery software JMP Pro version 13.2.0 (SAS Institute Japan Ltd., Tokyo) in which parity, litter size and their possible two-way interactions were treated as fixed effects and litter was treated as a random effect. The fixed effects and interaction effects that were significant at the nominal 5% level were included in the final model. Adjusted traits values were obtained by including body weight at the age of dissection in the final model. Residuals after removing significant fixed effects and a random effect were used for statistical analyses. Traits measured were classified into eight groups by category. MANOVA was performed for each of the eight trait groups to test significances for effects of sex, genotype, genotype-by-sex interactions and others. Phenotypic differences for individual traits of each group were determined by one-way ANOVA followed by Tukey’s HSD *post hoc* test. The four CIT component tests were performed with a linear model of JMP Pro.

## Electronic supplementary material

Supplementary information

## Data Availability

All data generated or analysed during this study are included in this published article and supplementary information. The DNA sequence data for a targeted genomic region of the KO strain have been deposited in DDBJ under the accession number of LC415908.

## References

[1] Fall T (2013). The role of adiposity in cardiometabolic traits: a Mendelian randomization analysis. PLoS Med..

[2] MacArthur J (2017). The new NHGRI-EBI Catalog of published genome-wide association studies (GWAS Catalog). Nucleic Acids Res..

[3] Locke AE (2015). Genetic studies of body mass index yield new insights for obesity biology. Nature.

[4] Smith CL (2018). The Mouse Genome Database (MGD)-2018: knowledgebase for the laboratory mouse. Nucleic Acids Res..

[5] Flint J, Valdar W, Shifman S, Mott R (2005). Strategies for mapping and cloning quantitative trait genes in rodents. Nat. Rev. Genet..

[6] Morton NM (2016). Genetic identification of thiosulfate sulfurtransferase as an adipocyte-expressed antidiabetic target in mice selected for leanness. Nat. Med..

[7] Ishikawa A, Matsuda Y, Namikawa T (2000). Detection of quantitative trait loci for body weight at 10 weeks from Philippine wild mice. Mamm. Genome.

[8] Ishikawa A, Hatada S, Nagamine Y, Namikawa T (2005). Further mapping of quantitative trait loci for postnatal growth in an intersubspecific backcross of wild *Mus musculus castaneus* and C57BL/6J mice. Genet. Res..

[9] Ishikawa A, Kim EH, Bolor H, Mollah MBR, Namikawa T (2007). A growth QTL (*Pbwg1*) region of mouse chromosome 2 contains closely linked loci affecting growth and body composition. Mamm. Genome.

[10] Mollah MBR, Ishikawa A (2010). A wild derived quantitative trait locus on mouse chromosome 2 prevents obesity. BMC Genet..

[11] Ishikawa A, Okuno S (2014). Fine mapping and candidate gene search of quantitative trait loci for growth and obesity using mouse intersubspecific subcongenic intercrosses and exome sequencing. PLoS ONE.

[12] Ishikawa A (2017). Identification of a putative quantitative trait gene for resistance to obesity in mice using transcriptome analysis and causal inference tests. PLoS ONE.

[13] Guo M (2000). A monoclonal antibody to the DEC-205 endocytosis receptor on human dendritic cells. Human Immunol..

[14] Rikke BA, Johnson TE (1998). Towards the cloning of genes underlying murine QTLs. Mamm. Genome.

[15] Mandard S, Müller M, Kersten S (2004). Peroxisome proliferator-activated receptor α target genes. CMLS, Cell. Mol. Life Sci..

[16] Millstein J, Zhang B, Zhu J, Schadt EE (2009). Disentangling molecular relationships with a causal inference test. BMC Genet..

[17] Ishikawa A (2017). A strategy for identifying quantitative trait genes using gene expression analysis and causal analysis. Genes.

[18] El-Brolosy MA, Stainier DYR (2017). Genetic compensation: a phenomenon in search of mechanisms. PLoS Genet..

[19] Sztal TE (2018). Genetic compensation triggered by actin mutation prevents the muscle damage caused by loss of actin protein. PLoS Genet..

[20] Cheverud JM (2010). Calpain-10 is a component of the obesity-related quantitative trait locus. Adip1. J. Lipid Res..

[21] Christians JK, de Zwaan DR, Fung SHY (2013). Pregnancy associated plasma protein A2 (PAPP-A2) affects bone size and shape and contributes to natural variation in postnatal growth in mice. PLoS ONE.

[22] Yalcin B (2004). Genetic dissection of a behavioral quantitative trait locus shows that *Rgs2* modulates anxiety in mice. Nat. Genet..

[23] Amanda H (2006). A locus on mouse chromosome 9 (*Adip5*) affects the relative weight of the gonadal but not retroperitoneal adipose depot. Mamm. Genome.

[24] Yamamoto Y (2010). Adipose depots possess unique developmental gene signatures. Obesity.

[25] Hu P, Jiao R, Jin L, Xiong M (2018). Application of causal inference to genomic analysis: advances in methodology. Front. Genet..

[26] Jiang W (1995). The receptor DEC-205 expressed by dendritic cells and thymic epithelial cells is involved in antigen processing. Nature.

[27] Boyle EA, Li YI, Pritchard JK (2017). An expanded view of complex traits: from polygenic to omnigenic. Cell.

