# Supplementary material for: Genetic identification of Ly75 as a novel quantitative trait gene for resistance to obesity in mice
Source: Sci Rep. 2018 Dec 5;8:17658. doi: 10.1038/s41598-018-36073-0 (PMC6281609; doi:10.1038/s41598-018-36073-0)
Supplement: Supplementary file 1 — Supplementary information [file 41598_2018_36073_MOESM1_ESM.pdf]

Supplementary information

Genetic identification of *Ly75* as a novel quantitative trait gene for resistance to obesity in mice

Keita Makino[1], Akira Ishikawa[1,*]

[1]Laboratory of Animal Genetics and Breeding, Graduate School of Bioagricultural Sciences, Nagoya University, Chikusa-ku, Nagoya 464-8601, Japan

*corresponding author A.I. (email: ishikawa@agr.nagoya-u.ac.jp)

Supplementary Tables

Table S1. Comparisons of trait measurements for male mice between the KO strain and its background B6J strain and between the SR24 strain and its background B6JJcl strain.

Trait	KO	B6J	*P* value	SR24	B6JJcl	*P* value
No. of mice	6	4		5	6	
Body weight at 6 weeks (g)	20.81±0.28	20.57±0.34	0.60	22.50±0.49	21.85±0.45	0.36
Body weight at 8 weeks (g)	22.65±0.26	22.06±0.32	0.20	24.65±0.31	23.89±0.28	0.10
Body weight at 12 weeks (g)	25.78±0.23	24.97±0.28	0.057	27.79±0.36	27.11±0.33	0.19
Body weight at 14 weeks (g)	25.98±0.31	25.53±0.38	0.39	28.66±0.24	28.12±0.22	0.13
Weight gain at 6-8 weeks (g)	1.83±0.29	1.49±0.36	0.48	2.15±0.25	2.04±0.23	0.75
Weight gain at 12-14 weeks (g)	0.198±0.18	0.565±0.22	0.24	0.87±0.29	1.02±0.27	0.73
Food intake at 6-8 weeks (g)	45.6±1.00	44.7±1.22	0.55	45.6±1.01	45.9±0.92	0.82
Food intake at 12-14 weeks (g)	50.3±1.44	52.6±1.76	0.36	54.9±1.25	54.0±1.14	0.60
Total body length (cm)	17.58±0.089	17.11±0.11	0.010	17.48±0.11	17.26±0.10	0.18
Head-body length (cm)	8.75±0.079	8.64±0.097	0.43	9.02±0.074	8.88±0.068	0.20
Tail length (cm)	8.83±0.057	8.47±0.069	0.0037	8.46±0.081	8.38±0.074	0.50
Total fat pad weight (g)	0.633±0.035	0.650±0.043	0.77	0.690±0.035	0.866±0.032	0.0047
Inguinal fat pad weight (g)	0.260±0.023	0.250±0.028	0.79	0.316±0.021	0.361±0.019	0.14
Gonadal fat pad weight (g)	0.373±0.016	0.400±0.019	0.32	0.374±0.017	0.505±0.016	0.00035

Liver weight (g)	1.38±0.029	1.25±0.036	0.028	1.40±0.057	1.47±0.053	0.37
Kidneys weight (g)	0.443±0.0078	0.375±0.0096	0.00053	0.492±0.0086	0.423±0.0078	0.00021
Heart weight (g)	0.127±0.0039	0.199±0.0048	0.25	0.160±0.0042	0.134±0.0039	0.0012
Lungs weight (g)	0.148±0.0056	0.144±0.0068	0.72	0.156±0.0056	0.165±0.0051	0.28
Testes weight (g)	0.201±0.0047	0.209±0.0058	0.34	0.216±0.0054	0.206±0.0049	0.20
Adjusted total body length	16.99±0.09	16.53±0.11	0.012	16.83±0.11	16.62±0.10	0.20
Adjusted head-body length	6.47±0.08	6.40±0.10	0.615	6.51±0.08	6.42±0.07	0.40
Adjusted tail length	10.52±0.06	10.13±0.07	0.0022	10.31±0.09	10.20±0.08	0.39
Adjusted total fat pad weight	-0.636±0.030	-0.598±0.037	0.44	-0.711±0.033	-0.508±0.030	0.0013
Adjusted inguinal fat pad weight	-0.501±0.022	-0.498±0.027	0.93	-0.524±0.020	-0.463±0.018	0.050
Adjusted gonadal fat pad weight	-0.135±0.012	-0.099±0.015	0.11	-0.187±0.016	-0.045±0.015	0.00012
Adjusted liver weight	0.0823±0.0238	-0.0221±0.0292	0.024	-0.0266±0.0533	0.0734±0.0487	0.20
Adjusted kidney weight	-0.0374±0.0063	-0.0981±0.0077	0.00028	-0.0388±0.0080	-0.0982±0.0073	0.00039
Adjusted heart weight	-0.0997±0.0035	-0.1033±0.0043	0.53	-0.0894±0.0046	-0.1113±0.0042	0.0069
Adjusted lung weight	0.0031±0.0049	0.0023±0.0060	0.93	-0.0029±0.0057	0.0088±0.0052	0.16
Adjusted testis weight	0.1705±0.0049	0.1787±0.0060	0.33	0.1815±0.0054	0.1720±0.0049	0.22

Data are mean ± S.E.M. Total fat pad weight is the sum of weights of inguinal and gonadal fat pads. Adjusted data were obtained by adjustment of the raw data by body weight at 16 weeks of age. The *P* value was obtained by t-test.

Supplementary Table S2. Trait measurements and results of quantitative complementation tests in a four-way cross population among KO, B6J, SR24 and B6JJcl strains.

	Genotype				*P* value
Trait	B6J/B6JJcl	B6J/SR	KO/B6JJcl	KO/SR	KO × QTL
No. of mice	79-81	88-90	89-91	84-90	
Body weight at 1 week (g)	4.95±0.06	4.85±0.06	4.80±0.06	4.82±0.06	0.31
Body weight at 3 weeks (g)	10.78±0.10	10.72±0.10	10.66±0.10	10.82±0.10	0.26
Body weight at 6 weeks (g)	22.90±0.16	23.07±0.15	22.66±0.15	23.07±0.15	0.44
Body weight at 10 weeks (g)	27.10±0.24	27.57±0.22	26.64±0.22	27.27±0.22	0.73
Body weight at 13 weeks (g)	34.19±0.37	34.16±0.35	33.38±0.35	34.40±0.35	0.14
Body weight at 16 weeks (g)	38.84±0.47[ab]	38.63±0.45[ab]	37.33±0.45[b]	39.07±0.45[a]	0.033
Weight gain at 1-3 weeks (g)	5.93±0.08	5.99±0.08	5.94±0.08	6.07±0.08	0.67
Weight gain at 3-6 weeks (g)	12.12±0.13	12.35±0.12	12.01±0.12	12.23±0.12	0.99
Weight gain at 6-10 weeks (g)	3.84±0.14	4.18±0.14	3.66±0.14	3.91±0.14	0.76
Weight gain at 10-13 weeks (g)	7.09±0.20	6.59±0.19	6.74±0.20	7.12±0.19	0.023
Weight gain at 13-16 weeks (g)	4.65±0.18[a]	4.47±0.17[ab]	3.95±0.17[b]	4.67±0.17[a]	0.0089
Total body length (cm)	17.49±0.03	17.54±0.03	17.43±0.03	17.53±0.03	0.45
Head-body length (cm)	9.25±0.07	9.34±0.06	9.28±0.06	9.34±0.06	0.83
Tail length (cm)	8.23±0.06	8.20±0.06	8.14±0.06	8.19±0.06	0.51
Total fat pad weight (g)	3.824±0.116	3.737±0.111	3.539±0.110	3.940±0.110	0.029

Inguinal fat pad weight (g)	1.912±0.061[ab]	1.857±0.056[ab]	1.750±0.057[a]	1.974±0.058[b]	0.018
Gonadal fat pad weight (g)	1.913±0.057	1.880±0.054	1.790±0.054	1.966±0.054	0.057
Liver weight (g)	1.350±0.021	1.378±0.020	1.301±0.020	1.345±0.020	0.69
Kidney weight (g)	0.430±0.004	0.431±0.004	0.422±0.004	0.431±0.004	0.23
Heart weight (g)	0.138±0.001	0.139±0.001	0.136±0.001	0.138±0.001	0.75
Lung weight (g)	0.155±0.001	0.158±0.001	0.154±0.001	0.156±0.001	0.57
Spleen weight (g)	0.111±0.002	0.109±0.002	0.109±0.002	0.109±0.002	0.61
Testis weight (g)	0.232±0.003	0.232±0.002	0.226±0.002	0.227±0.002	0.66
Adjusted total body length	-0.023±0.027	0.035±0.026	-0.019±0.025	0.043±0.256	0.50
Adjusted head-body length	-0.069±0.0610	0.024±0.058	0.037±0.058	0.036±0.579	0.28
Adjusted tail length	0.046±0.059	0.011±0.056	-0.056±0.055	0.007±0.559	0.42
Adjusted total fat pad weight	-0.008±0.048	-0.053±0.047	0.012±0.045	0.061±0.046	0.31
Adjusted inguinal fat pad weight	0.004±0.028	-0.030±0.027	-0.008±0.027	0.044±0.027	0.11
Adjusted gonadal fat pad weight	-0.013±0.024	-0.023±0.023	0.020±0.022	0.018±0.023	0.86
Adjusted liver weight	-0.006±0.011[ab]	0.029±0.011[a]	-0.003±0.011[ab]	-0.018±0.011[b]	0.018
Adjusted kidney weight	-0.00050±0.00357	0.00063±0.00341	0.00028±0.00332	-0.00116±0.00350	0.71
Adjusted heart weight	-0.00073±0.00102	0.00069±0.00097	0.00040±0.00097	-0.00061±0.00097	0.22
Adjusted lung weight	-0.0012±0.0011	0.0019±0.0011	-0.0006±0.0010	-0.0002±0.0011	0.20
Adjusted spleen weight	0.0018±0.0017	-0.0002±0.0016	-0.0008±0.0016	-0.0003±0.0016	0.46
Adjusted testis weight	0.0029±0.0024	0.0022±0.0019	-0.0013±0.0022	-0.0032±0.0019	0.77

Sex-combined data are presented as the least squares mean ± S.E.M. adjusted for sex and litter effects. Total fat pad weight is the sum of weights of inguinal and gonadal white fat pads. Adjusted data were obtained by adjustment of the raw data by sex and litter effects and body weight at 16 weeks of age. For the quantitative complementation test, two-way ANOVA was used to test an interaction effect between KO and QTL alleles (see Fig. 1a). [a,b]Least squares means with different superscript letters within a trait indicate significant differences among mice with the four genotypes at $P < 0.05$ (Tukey's honestly significant difference (HSD) test).

Supplementary Table S3. Results of MANOVA for eight trait groups in a four-way cross population among KO, B6J, SR24 and B6JJcl strains.

Model effect	Test	Body weight	Weight gain	Body length	Organ weight	Fat weight	Adjusted body length	Adjusted organ weight	Adjusted fat weight
No. of mice		333	333	352	347	352	352	347	352
No. of traits		6	5	2	5	2	2	5	2
Whole model	Wilks' Lambda	1.6E-50	1.3E-52	8.1E-21	2.3E-53	1.6E-36	0.49	5.6E-07	5.4E-05
	Pillai's Trace	1.3E-32	4.1E-35	8.3E-19	2.5E-34	2.1E-31	0.49	1.3E-06	6.1E-05
	Hotelling-Lawley Trace	8.1E-70	2.5E-70	3.0E-22	2.1E-73	5.0E-40	0.49	3.3E-07	5.2E-05
	Roy's Maximum Root	1.9E-64	1.2E-63	5.4E-24	2.6E-67	2.1E-39	0.30	2.1E-10	1.1E-05
KO	F-test	0.097	0.16	0.44	0.31	0.88	0.69	0.22	0.26
QTL	F-test	0.040	0.021	0.078	0.16	0.40	0.28	0.31	0.74
KO×QTL	F-test	0.087	0.068	0.53	0.52	0.032	0.62	0.020	0.13
Sex	F-test	9.5E-62	5.2E-62	7.0E-25	4.0E-66	1.8E-41	0.61	2.2.E-10	7.1.E-07
KO×Sex	F-test	0.73	0.60	0.21	0.49	0.71	0.49	0.72	0.43
QTL×Sex	F-test	0.57	0.42	0.45	0.71	0.11	0.11	0.29	0.05
KO×QTL×Sex	F-test	0.97	0.95	0.46	0.92	0.47	0.45	0.92	0.58

Supplementary Table S4. Trait measurements in an F_2 intercross population between KO and B6J strains.

Trait	Genotype			*P* value
	KO/KO	KO/B6J	B6J/B6J	
No. of mice	34	44	30	
Body weight at 1 week (g)	4.04±0.05	4.11±0.04	4.13±0.05	0.45
Body weight at 3 weeks (g)	8.42±0.13	8.67±0.11	8.49±0.13	0.28
Body weight at 6 weeks (g)	20.05±0.24	20.03±0.21	20.45±0.25	0.39
Body weight at 10 weeks (g)	24.97±0.40	25.35±0.34	25.67±0.41	0.47
Body weight at 13 weeks (g)	28.40±0.54	29.14±0.47	29.36±0.56	0.42
Body weight at 16 weeks (g)	31.11±0.67	31.97±0.57	32.05±0.70	0.53
Weight gain at 1-3 weeks (g)	4.56±0.09	4.56±0.08	4.35±0.09	0.15
Weight gain at 3-6 weeks (g)	11.63±0.22	11.36±0.19	11.96±0.23	0.13
Weight gain at 6-10 weeks (g)	4.92±0.22	5.32±0.19	5.22±0.23	0.37
Weight gain at 10-13 weeks (g)	3.43±0.22	3.78±0.19	3.69±0.22	0.44
Weight gain at 13-16 weeks (g)	2.72±0.19	2.84±0.16	2.69±0.19	0.81
Total body length (cm)	17.29±0.04	17.36±0.04	17.43±0.05	0.095
Head-body length (cm)	9.11±0.03	9.17±0.03	9.19±0.04	0.22
Tail length (cm)	8.18±0.02	8.18±0.02	8.24±0.03	0.16
Total fat pad weight (g)	3.502±0.205	3.919±0.177	3.681±0.214	0.30
Inguinal fat pad weight (g)	1.589±0.093	1.785±0.080	1.648±0.097	0.25
Gonadal fat pad weight (g)	1.248±0.077	1.382±0.066	1.341±0.080	0.40
Perirenal fat pad weight (g)	0.665±0.039	0.752±0.034	0.693±0.041	0.22
Liver weight (g)	1.038±0.024	1.018±0.021	1.040±0.025	0.75
Kidney weight (g)	0.324±0.005	0.322±0.005	0.338±0.006	0.85
Heart weight (g)	0.108±0.002	0.109±0.001	0.113±0.002	0.066
Lung weight (g)	0.137±0.002[a]	0.139±0.002[ab]	0.144±0.002[b]	0.031
Spleen weight (g)	0.080±0.002[a]	0.079±0.002[a]	0.088±0.002[b]	0.0020
Testis weight (g)	0.186±0.011	0.194±0.011	0.209±0.013	0.39
Adjusted total body length	-0.039±0.032	-0.011±0.028	0.060±0.034	0.094
Adjusted head-body length	-0.022±0.019	0.004±0.017	0.019±0.021	0.34
Adjusted tail length	-0.017±0.024	-0.015±0.021	0.041±0.025	0.17
Adjusted total fat pad weight	-0.052±0.072[ab]	0.130±0.063[a]	-0.131±0.036[b]	0.024
Adjusted inguinal fat pad weight	-0.024±0.034[ab]	0.071±0.030[a]	-0.077±0.037[b]	0.0070

Adjusted gonadal fat pad weight	-0.015±0.030	0.027±0.027	-0.022±0.032	0.42
Adjusted perirenal fat pad weight	-0.013±0.016[ab]	0.032±0.014[a]	-0.032±0.017[b]	0.0096
Adjusted liver weight	0.026±0.0114[a]	-0.020±0.010[b]	-0.0004±0.012[ab]	0.015
Adjusted kidney weight	0.0011±0.0040[ab]	-0.0060±0.0035[a]	0.0086±0.0043[b]	0.023
Adjusted heart weight	-0.0008±0.0011[ab]	-0.0012±0.0010[a]	0.0027±0.0012[b]	0.038
Adjusted lung weight	-0.0026±0.0018[a]	-0.0007±0.0016[ab]	0.0040±0.0019[b]	0.039
Adjusted spleen weight	-0.0022±0.0019[a]	-0.0026±0.0017[a]	0.0063±0.0020[b]	0.0019
Adjusted testis weight	-0.0074±0.0100	0.0002±0.0096	0.0100±0.012	0.53

Sex-combined data are presented as the least squares mean ± S.E.M. adjusted for sex and litter effects. Total fat pad weight is the sum of weights of inguinal, gonadal and perirenal fat pads. Adjusted data were obtained by adjustment of the raw data by sex and litter effects and body weight at 16 weeks of age. [a,b]Least squares means with different superscript letters within a trait indicate significant differences among mice with the three genotypes at $P < 0.05$ (one-way ANOVA folowed by Tukey's HSD test).

Supplementary Table S5. Results of MANOVA for eight trait groups in an F_2 intercross population between KO and B6J strains.

Model effect	Test	Body weight	Weight gain	Body length	Organ weight	Fat weight	Adjusted body length	Adjusted organ weight	Adjusted fat weight
No. of mice		108	108	108	108	108	108	108	108
No. of traits		6	5	2	5	4	2	5	4
Whole model	Wilks' Lambda	0.0055	0.0064	0.0090	7.5E-04	0.012	0.17	0.0065	0.13
	Pillai's Trace	0.0087	0.011	0.011	0.0010	0.014	0.17	0.0061	0.15
	Hotelling-Lawley Trace	0.0044	0.0044	0.0084	8.6E-04	0.011	0.17	0.0087	0.12
	Roy's Maximum Root	9.1E-06	3.5E-06	0.0012	3.1E-05	5.5E-04	0.056	0.0030	0.0027
Genotype	Wilks' Lambda	0.21	0.37	0.086	0.039	0.29	0.074	0.0023	0.076
	Pillai's Trace	0.22	0.37	0.086	0.041	0.29	0.074	0.0022	0.082
	Hotelling-Lawley Trace	0.21	0.36	0.088	0.040	0.30	0.076	0.0029	0.072
	Roy's Maximum Root	0.052	0.093	0.042	0.010	0.10	0.032	0.0068	0.0098
Sex	F-test	1.4E-04	5.7E-05	4.3E-04	7.6E-05	2.3E-04	0.81	0.19	0.17
Genotype×Sex	Wilks' Lambda	0.63	0.84	0.41	0.74	0.95	0.078	0.64	0.77
	Pillai's Trace	0.62	0.84	0.41	0.74	0.95	0.080	0.64	0.76
	Hotelling-Lawley Trace	0.63	0.84	0.41	0.74	0.95	0.076	0.64	0.77
	Roy's Maximum Root	0.38	0.55	0.14	0.32	0.66	0.015	0.31	0.46

Supplementary Table S6. Trait measurements for F_2 male mice obtained from an intercross between KO and SR24 strains.

Trait	Genotype			*P* value
	KO/KO	KO/SR	SR/SR	
No. of mice	25-26	35-38	27	
Food intake at 4-6 weeks (g)	46.71±1.87	47.84±1.53	47.49±1.62	0.90
Body weight at 1 week (g)	4.14±0.05	4.13±0.04	4.05±0.05	0.29
Body weight at 3 weeks (g)	8.87±0.12	8.72±0.10	8.86±0.12	0.56
Body weight at 6 weeks (g)	21.49±0.19	21.63±0.16	21.66±0.19	0.79
Body weight at 10 weeks (g)	32.27±0.29	32.65±0.33	32.27±0.40	0.68
Body weight at 13 weeks (g)	28.40±0.54	29.14±0.47	29.36±0.56	0.42
Body weight at 16 weeks (g)	33.80±0.43	34.52±0.35	34.36±0.42	0.42
Weight gain at 1-3 weeks (g)	4.72±0.10	4.59±0.08	4.81±0.10	0.21
Weight gain at 3-6 weeks (g)	12.62±0.19	12.90±0.13	12.80±0.15	0.37
Weight gain at 6-10 weeks (g)	6.31±0.19	6.33±0.15	5.87±0.18	0.12
Weight gain at 10-13 weeks (g)	4.47±0.20	4.69±0.16	4.74±0.19	0.57
Weight gain at 13-16 weeks (g)	1.53±0.22	1.87±0.18	2.09±0.21	0.18
Total body length (cm)	17.56±0.04	17.67±0.04	17.57±0.05	0.19
Head-body length (cm)	9.21±0.03	9.30±0.03	9.24±0.03	0.071
Tail length (cm)	8.36±0.03	8.37±0.03	8.33±0.03	0.60
Total fat pad weight (g)	3.889±0.139	4.000±0.118	3.759±0.134	0.41
Inguinal fat pad weight (g)	1.855±0.074	1.955±0.061	1.926±0.072	0.58
Gonadal fat pad weight (g)	1.338±0.056	1.371±0.047	1.224±0.055	0.12
Perirenal fat pad weight (g)	0.690±0.030	0.698±0.025	0.626±0.029	0.14
Liver weight (g)	1.177±0.026	1.207±0.022	1.239±0.026	0.25
Kidney weight (g)	0.384±0.004[a]	0.392±0.004[ab]	0.400±0.004[b]	0.028
Heart weight (g)	0.113±0.001[a]	0.116±0.001[ab]	0.117±0.001[b]	0.029
Lung weight (g)	0.145±0.002	0.146±0.001	0.147±0.002	0.66
Spleen weight (g)	0.078±0.001	0.081±0.001	0.082±0.001	0.084
Testis weight (g)	0.218±0.003	0.221±0.002	0.215±0.003	0.25
Adjusted total body length	-0.016±0.045	0.044±0.037	-0.047±0.044	0.27
Adjusted head-body length	-0.027±0.026	0.033±0.021	-0.020±0.025	0.13
Adjusted tail length	0.011±0.004	0.011±0.026	-0.027±0.030	0.57

Adjusted total fat pad weight	0.109±0.069[a]	0.045±0.059[ab]	-0.160±0.067[b]	0.015
Adjusted inguinal fat pad weight	0.004±0.041	0.001±0.034	-0.006±0.040	0.98
Adjusted gonadal fat pad weight	0.061±0.041[a]	0.031±0.034[a]	-0.102±0.040[b]	0.011
Adjusted perirenal fat pad weight	0.035±0.022[a]	0.015±0.019[ab]	-0.052±0.022[b]	0.015
Adjusted liver weight	-0.008±0.017	-0.013±0.014	0.027±0.016	0.150
Adjusted kidney weight	-0.0068±0.0041[a]	-0.0008±0.0034[ab]	0.0077±0.0040[b]	0.042
Adjusted heart weight	-0.0020±0.0010	0.00029±0.00085	0.00148±0.00100	0.057
Adjusted lung weight	-0.00052±0.00158	-0.00031±0.00130	0.00095±0.00155	0.76
Adjusted spleen weight	-0.0057±0.0014	0.0004±0.0012	0.0018±0.0014	0.097
Adjusted testis weight	0.0003±0.0028	0.0025±0.0024	-0.0034±0.0027	0.26

Food intake was measured in only males (n = 6 for KO/KO, 9 for KO/SR, 8 for SR/SR). Sex-combined data are presented as the least squares mean ± S.E.M. adjusted for a litter effect. Total fat pad weight is the sum of weights of inguinal, gonadal and perirenal fat pads. Adjusted data were obtained by adjustment of the raw data by a litter effect and body weight at 16 weeks of age. [a,b]Least squares means with different superscript letters within a trait indicate significant differences among mice with the three genotypes at $P < 0.05$ (one-way ANOVA followed by Tukey's HSD test).

Supplementary Table S7. Results of MANOVA for eight trait groups in F_2 male mice obtained from an intercross between KO and SR24 strains.

Model effect	Test	Body weight	Weight gain	Body length	Organ weight	Fat weight	Adjusted body length	Adjusted organ weight	Adjusted fat weight
No. of mice		91	91	91	87	87	91	87	87
No. of traits		6	5	2	6	4	2	6	4
Whole model	Wilks' Lambda	0.13	0.13	0.19	0.040	0.34	0.29	0.025	0.11
	Pillai's Trace	0.13	0.13	0.19	0.039	0.34	0.29	0.025	0.11
	Hotelling-Lawley Trace	0.14	0.14	0.19	0.043	0.34	0.29	0.027	0.10
	Roy's Maximum Root	0.047	0.07	0.069	0.023	0.11	0.13	0.012	0.021
Genotype	Wilks' Lambda	0.13	0.13	0.19	0.040	0.34	0.29	0.025	0.11
	Pillai's Trace	0.13	0.13	0.19	0.039	0.34	0.29	0.025	0.11
	Hotelling-Lawley Trace	0.14	0.14	0.19	0.043	0.34	0.29	0.027	0.10
	Roy's Maximum Root	0.047	0.067	0.069	0.023	0.11	0.13	0.012	0.021

Supplementary Table S8. Tissue expression of *Ly75* in KO/B6J and B6J/B6J F_2 male mice obtained from an intercross between KO and B6J strains.

	Genotype			
Tissue	KO/B6J	B6J/B6J	Fold change	*P* value
No. of mice	3	3		
Inguinal fat pad	4.21±2.72	14.46±6.21	3.4	0.36
Gonadal fat pad	0.13±0.041	0.14±0.02	1.1	0.93
Diencephalon	0.33±0.02	0.72±0.08	2.2	0.037
Pituitary gland	1.49±0.04	3.05±0.00	2.0	0.0017
Thymus	7.16±0.84	10.67±2.66	1.5	0.43
Liver	1.00±0.08	1.99±0.27	2.0	0.070
Spleen	1.15±0.22	2.80±0.49	2.4	0.10
Kidney	0.73±0.12	1.25±0.288	1.7	0.32
Ileum	1.39±0.21	2.79±0.38	2.0	0.093
Gastrocnemius muscle	0.09±0.01	0.29±0.03	3.2	0.016

Pituitary glands were measured for two mice per genotype. Data are mean ± S.E.M. for expression levels relative to the KO/B6J liver. The fold change is the ratio of B6J/B6J to KO/B6J. The *P* value was obtained by t-test.

Supplementary Table S9. Primer pairs used in this study.

Locus	Purpose		Primer (5'-3')
D2Mit123	Genotyping of a subcongenic region	Forward	TGTGTCTTTTATTAATTGTCTCCTCC
		Reverse	GGAAATCCTTGGTTGCAGTC
rs48690987	Genotyping of a subcongenic region	Forward	AAATTCATCCGTTTCGTCCA
		Reverse	GGATAGTTTTCTGCCCTTTGC
Ly75	Genotyping of the KO allele	IMR0162	CCGGTTCTTTTTGTCAAGACCG
		IMR0163	CGGCAGGAGCAAGGTGAGAT
Ly75	Genotyping of the wild-type allele	IMR3919	AAACCCTGTGGTTGTCCTGT
		IMR3920	GCATGATTTCAGGGGACAGT
Actb	Quantitative real-time PCR	Forward	GGCTGTATTCCCCTCCATCG
		Reverse	CCAGTTGGTAACAATGCCATGT
Ly75	Quantitative real-time PCR	Forward	GGCTTGTGGAGCCTTCTGAGA
		Reverse	GCCAGTGTTTTCATGGACGAT
Ly75	Sequencing	Forward	TGCTCCTGCCGAGAAAGTAT
		Reverse	TCACATTTCCACCACAGCAT

Supplementary Figures

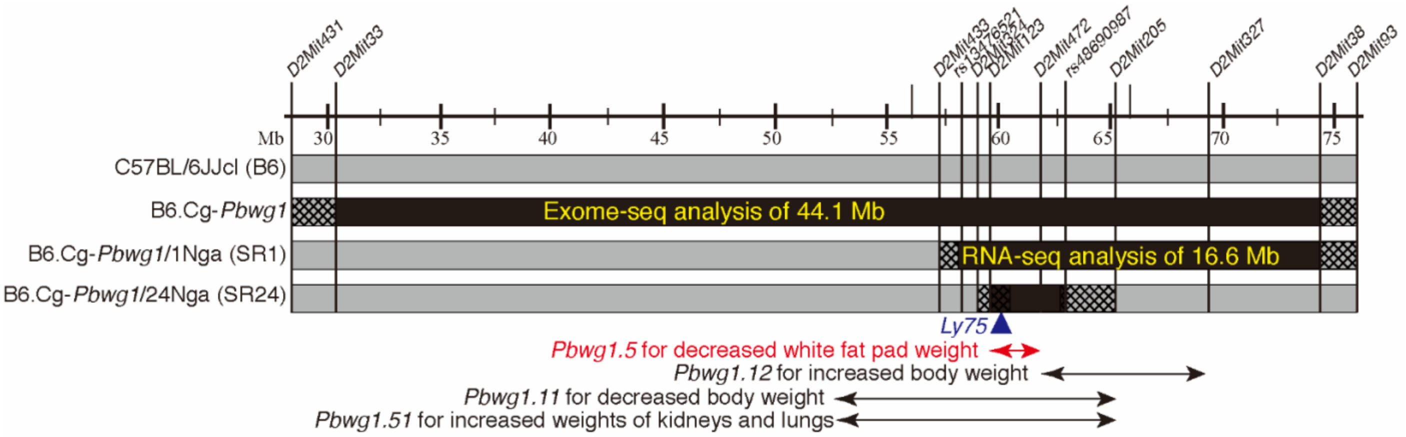

Supplementary Fig. S1. Genomic regions of subcongenic strains (B6.Cg-*Pbwg1*/#Nga, abbreviation: SR#) created from the original B6.Cg-*Pbwg1* congenic strain carrying the *Pbwg1* QTL on mouse chromosome 2. The black and grey bars indicate genomic intervals derived from wild *Mus musculus castaneus* and C57BL/6JJcl (B6JJcl) mice, respectively. The hatched regions indicate intervals where recombination occurred. Approximate map positions in mega base pairs (Mb) for DNA markers (*D2Mit#* and *rs#*) are shown on the horizontal line. Exome-seq and RNA-seq analyses were previously performed on the wild-derived intervals of the original congenic strain[1] and the SR1 strain[2], respectively. The blue triangle indicates the position of the *lymphocyte antigen 75* (*Ly75*) gene, a putative QTG for *Pbwg1.5*[2]. The double-headed arrows indicate the maximum intervals of QTLs for body weight (*Pbwg1.11* and *Pbwg1.12*)[3], white fat pad weight (*Pbwg1.5*) and weights of kidneys and lungs (named *Pbwg1.51* in the present study)[4]. The effects of the wild-derived allele at these QTLs are shown near the double-headed arrows.

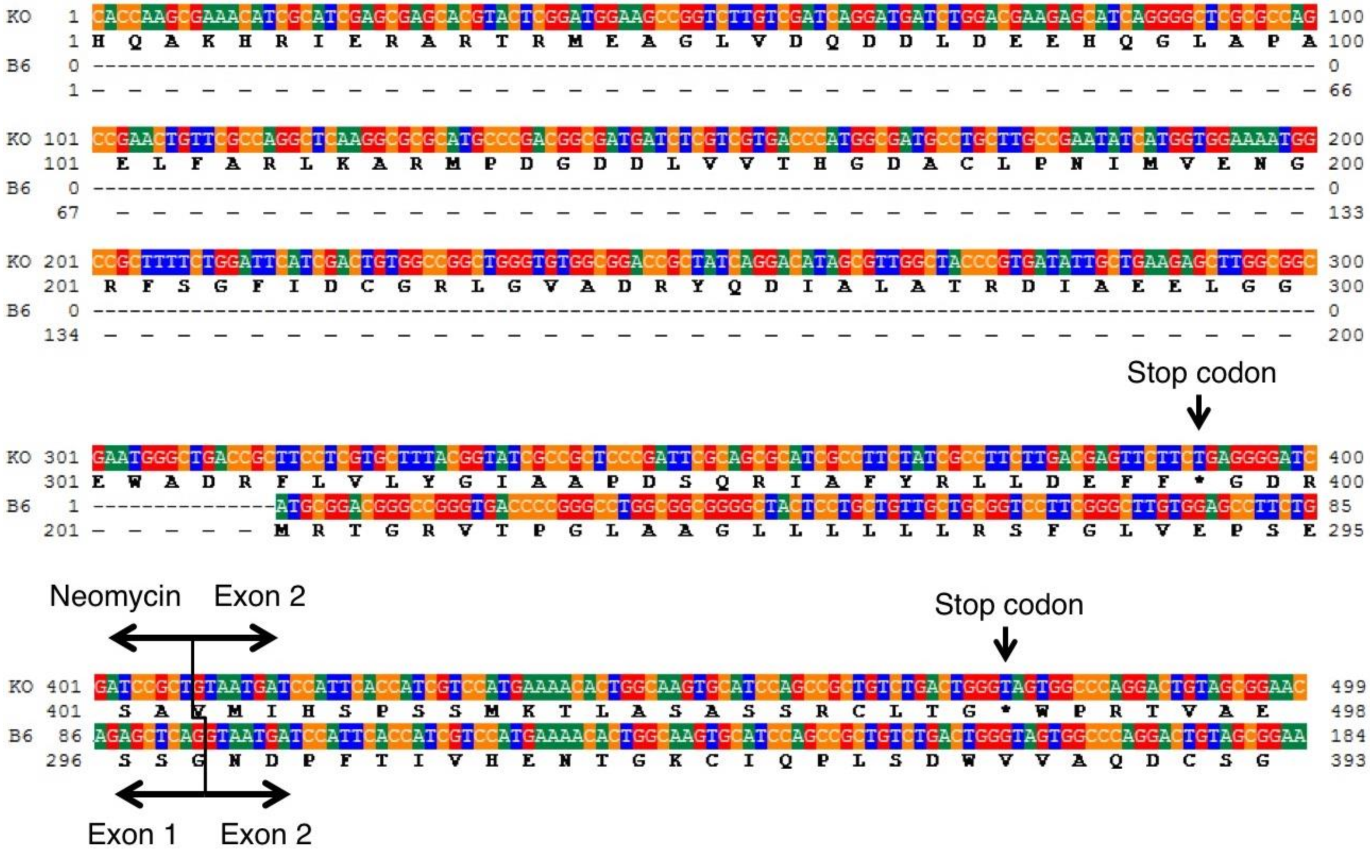

Supplementary Fig. S2. DNA sequence analysis of the targeted genomic region of the B6.129P-*Ly75*$^{tm/Mnz}$/J (KO) strain knocked out for the *Ly75* gene. The upper sequence is the sequence of the KO strain, and the lower sequence is the reference sequence of C57BL/6J (RefSeq mm10). In the KO sequence, the sequence of a neomycin resistance gene was inserted just before *Ly75* exon 2 and thereby a frameshift mutation occurred, leading to the creation of a new stop codon in *Ly75* exon 2. The DNA sequence data for the KO strain have been deposited in DDBJ under the accession number of LC415908.

References

1. Ishikawa, A. & Okuno, S. Fine mapping and candidate gene search of quantitative trait loci for growth and obesity using mouse intersubspecific subcongenic intercrosses and exome sequencing. *PLoS ONE* **9**, e113233 (2014).
2. Ishikawa, A. Identification of a putative quantitative trait gene for resistance to obesity in mice using transcriptome analysis and causal inference tests. *PLoS ONE* **12**, e0170652 (2017).
3. Mollah, M. B. R. & Ishikawa, A. Intersubspecific subcongenic mouse strain analysis reveals closely linked QTLs with opposite effects on body weight. *Mamm. Genome* **22**, 282-289 (2011).
4. Mollah, M. B. R. & Ishikawa, A. Fine mapping of quantitative trait loci affecting organ weights by mouse intersubspecific subcongenic strain analysis. *Anim. Sci. J.* **84**, 296-302 (2013).
